# Survey of low pathogenic avian influenza viruses in live poultry markets in Guangxi Province, Southern China, 2016–2019

**DOI:** 10.1038/s41598-021-02639-8

**Published:** 2021-12-01

**Authors:** Sisi Luo, Zhixun Xie, Meng Li, Dan Li, Liji Xie, Jiaoling Huang, Minxiu Zhang, Tingting Zeng, Sheng Wang, Qing Fan, Yanfang Zhang, Zhiqin Xie, Xianwen Deng, Jiabo Liu

**Affiliations:** Guangxi Key Laboratory of Veterinary Biotechnology, Guangxi Veterinary Research Institute, 51 North Road You Ai, Nanning, 530001 Guangxi China

**Keywords:** Microbiology, Virology

## Abstract

Low pathogenic avian influenza viruses (LPAIVs) have been widespread in poultry and wild birds throughout the world for many decades. LPAIV infections are usually asymptomatic or cause subclinical symptoms. However, the genetic reassortment of LPAIVs may generate novel viruses with increased virulence and cross-species transmission, posing potential risks to public health. To evaluate the epidemic potential and infection landscape of LPAIVs in Guangxi Province, China, we collected and analyzed throat and cloacal swab samples from chickens, ducks and geese from the live poultry markets on a regular basis from 2016 to 2019. Among the 7,567 samples, 974 (12.87%) were LPAIVs-positive, with 890 single and 84 mixed infections. Higher yearly isolation rates were observed in 2017 and 2018. Additionally, geese had the highest isolation rate, followed by ducks and chickens. Seasonally, spring had the highest isolation rate. Subtype H3, H4, H6 and H9 viruses were detected over prolonged periods, while H1 and H11 viruses were detected transiently. The predominant subtypes in chickens, ducks and geese were H9, H3, and H6, respectively. The 84 mixed infection samples contained 22 combinations. Most mixed infections involved two subtypes, with H3 + H4 as the most common combination. Our study provides important epidemiological data regarding the isolation rates, distributions of prevalent subtypes and mixed infections of LPAIVs. These results will improve our knowledge and ability to control epidemics, guide disease management strategies and provide early awareness of newly emerged AIV reassortants with pandemic potential.

## Introduction

Avian influenza viruses (AIVs) are type A influenza viruses and belong to the *Orthomyxoviridae* family^1^. AIV is a zoonotic pathogen and can threaten the health of humans and animals^2^. AIV is an enveloped, single-stranded, negative-sense, segmented RNA virus^3^. The genome consists of eight gene segments: basic polymerase 2 (PB2), basic polymerase 1 (PB1), acidic polymerase (PA), hemagglutinin (HA), nucleoprotein (NP), neuraminidase (NA), matrix (M), and nonstructural (NS)^4^. AIVs are subtyped based on the antigenic diversity of two surface glycoproteins: HA and NA. Differences in the antigenicity and phylogenetics of these surface proteins allow characterization of AIV into subtypes H(x)N(y). To date, 16 HA (H1-H16) and 9 NA (N1-N9) subtypes have been recognized as circulating virus strains in poultry or wild birds, whereas H17N10 and H18N11were newly discovered in bats in recent years^5,6^. Highly pathogenic AIVs (HPAIVs) and low pathogenic AIVs (LPAIVs) are classified based on virus pathogenicity in poultry^7,8^. HPAIVs can cause severe disease outbreaks in poultry, resulting in heavy economic losses and posing serious public health concerns^9^. A few H5 and H7 subtypes are virulent HPAIVs and lead to high morbidity and mortality in poultry^10,11^. Most AIVs are LPAIVs. LPAIV strains, including the H1-H16 subtypes, and their infections are usually asymptomatic or induce subclinical signs of illness, and the infected animals appear to be healthy or exhibit only mild respiratory disease symptoms with low mortality^12^. LPAIVs may be silent in wild birds or poultry, but they could pose a risk to human health. For example, H7N9 emerged in the Yangtze River Delta in the spring of 2013 and caused five waves of infections until 2017. Although the H7N9 AIV had low pathogenicity in poultry at the beginning of the outbreak, it became an apparent public health issue due to increasing human mortality, and severely affected public health and socioeconomic development^13^.

The RNA polymerase in AIV lacks proofreading capacity of the eight genes^14^. Genetic reassortment may occur when two or more AIV subtypes coinfect a single cell, leading to the generation of novel AIV viruses with pandemic potential^15^. The virulences of novel viruses are unpredictable and may gain the potential to infect humans or different species. It was also reported in recent years that the H6, H9 and H10 subtypes of LPAIVs could cause zoonotic infections. The first human case of H10N8 infection was confirmed on December 17, 2013, and two other cases were subsequently confirmed, but no further outbreak and occurred, suggesting that H10N8 may be sporadic^16^. The H6N1 virus presented the first case of human infection in Taiwan in June 2013^17^. The H9N2 virus provided six internal genes to the emerging H7N9 and H10N8 responsible for human infection in 2013^16,18,19^. LPAIVs are usually not undiagnosed, evolve continuously and spread in natural hosts. Monitoring LPAIVs is useful not only for public health to prevent AIVs from spreading to humans, but also to monitor circulation in poultry and virus evolution.

Southern China has been considered an epicenter of influenza virus due to its poultry breeding and trading style. Guangxi Province is located in the southern part of China. Poultry breeding is extensive and booming in Guangxi Province; there are many large-scale farms and free range backyards, and more than 1.2 billion birds are raised annually. Chicken, duck and goose comprise the primary species in poultry production in Guangxi Province, and the output value is more than Ұ20 billion. In Guangxi Province, chicken farming is the most abundant, and yellow meat chicken is widely consumed throughout the country; Some chicken farms are located on hillsides, where chickens are raised in a free-range mode. Ducks farming is less common than chicken farming, but still accounts for a large number of birds raised; ducks were raised in rivers, in ponds and on seasides farms. Compared to chickens and ducks, geese production occurs on a smaller scale. Guangxi’s seaside cities Beihai and Fangcheng along the Beibu Gulf provide a good resting area for migrating wild birds. In addition, Guangxi Province neighbors Southeast Asian countries and borders Vietnam. Therefore, epidemiological investigation of LPAIVs in Guangxi province is important. At present, comprehensive epidemiological surveillance of LPAIVs in poultry in this region is scarce. Poultry in southern China is mainly traded through live poultry markets (LPMs), where different poultry species are housed together for several days, providing a favorable environment for virus transmission and recombination^20,21^. There are many LPMs in Guangxi, and the source of poultry in LPMs derived from different farms and free-fenced backyards in the province may vary greatly. Human infections with H5N1, H9N2 and recent H7N9 viruses were associated with exposure to LPMs^22–24^. Continued surveillance of poultry may generate valuable knowledge to support AIV prevention and control. We previously reported on the surveillance of LPAIVs in LPMs in Guangxi from 2009 to 2015^25,26^. In this study, we conducted an LPAIV surveillance study on chickens, ducks and geese in LPMs in Guangxi from 2016 to 2019, and found that LPAIVs exhibited an infection landscape over four-year periods.

## Results

### LPAIV isolation rates

**Figure 1:**
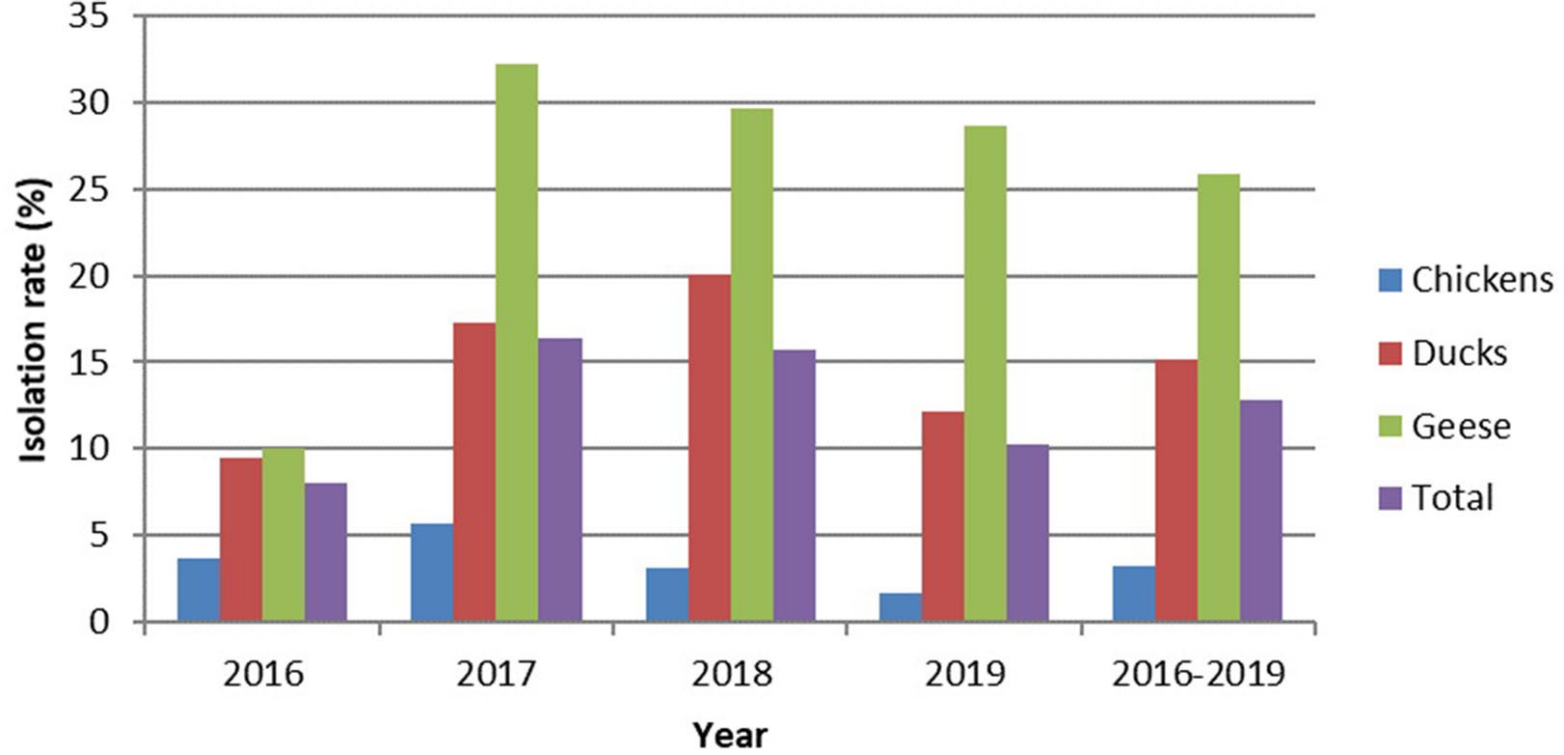
Yearly isolation rates of LPAIVs in chickens, ducks and geese from 2016 to 2019.

We collected a total of 7,567 swab samples from 2,175 chickens, 4,601 ducks and 791 geese in the LPMs of Guangxi Province from January 2016 to December 2019. Among the 7,567 samples, 974 (12.87%) samples were isolated and tested positive for LPAIVs. The yearly isolation rates were 8.00% (113/1413), 16.40% (266/1622), 15.75% (370/2349) and 10.31% (225/2183) from 2016 to 2019, respectively. Higher yearly isolation rates were observed in 2017 and 2018 (Fig. 1). Compared to that in 2016, the isolation rate was twice as high in 2017 (p < 0.001), decreased slightly in 2018, and continued to decrease in 2019 (p < 0.001).

Among the 974 LPAIV-positive samples, 71 were derived from chickens, 698 were derived from ducks and 205 were derived from geese. The isolation rates of chickens, ducks and geese were 3.26% (71/2175), 15.17% (698/4601) and 25.92% (205/791), respectively. Geese had the highest rate of virus isolation, followed by ducks and chickens (p < 0.001). The annual isolation rates of these poultry species from 2016 to 2019 are exhibited in Fig. 1. The isolation rate of ducks increased annually from 2016 to 2018 but significantly deceased in 2019 (p < 0.001). The isolation rates of chickens and geese both increased until peaking in 2017 and then decreased annually from 2018 to 2019.

**Figure 2:**
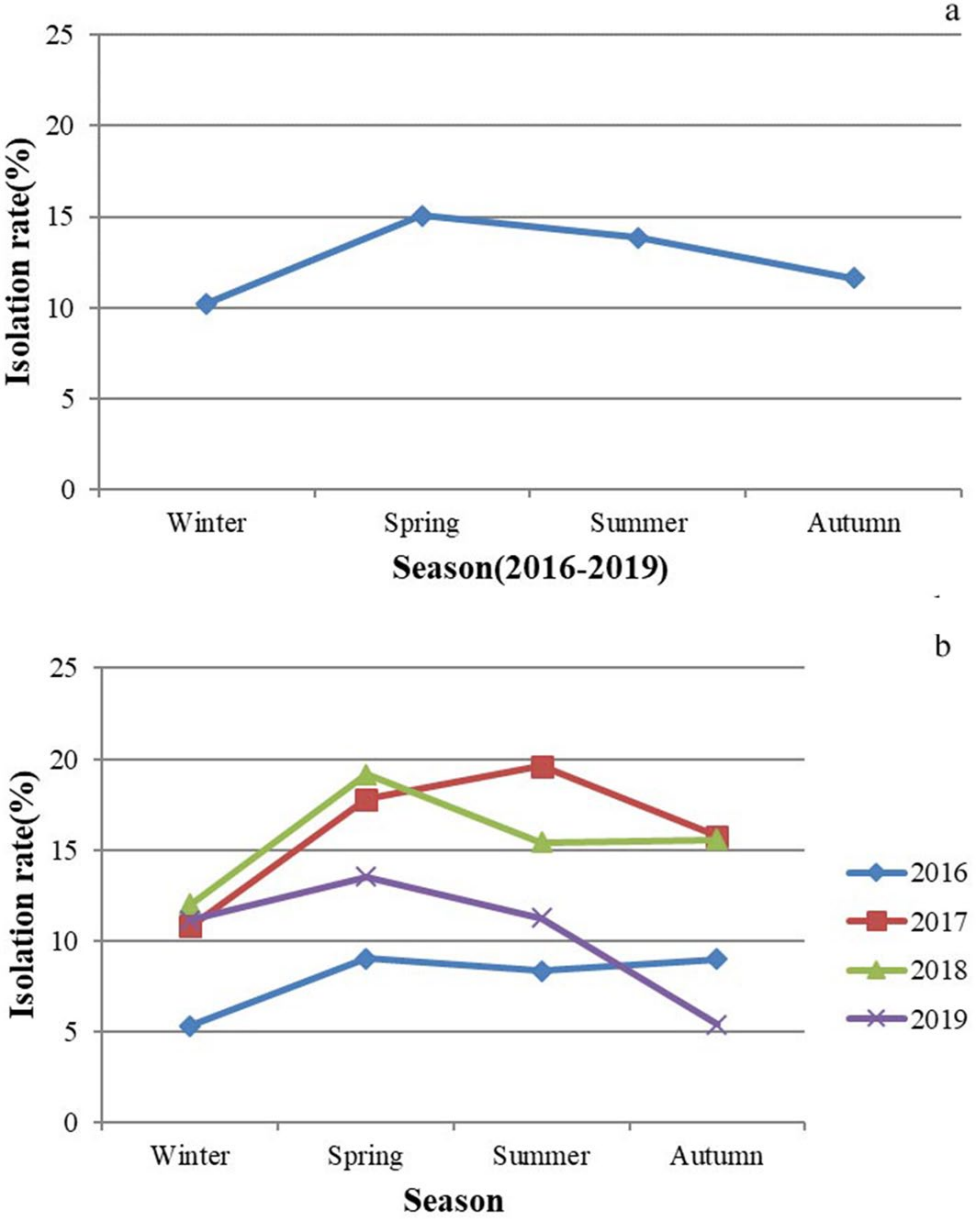
LPAIVs seasonal isolation rates. (a) The average isolation rates in the winter, spring, summer and autumn for four years from 2016 to 2019; (b)The seasonal isolation rates in each year from 2016 to 2019.

The average seasonal isolation rates for the four years are presented in Fig. 2a. Spring and summer had higher isolation rates, whereas the isolation rates of winter and autumn were relatively low (p < 0.05). The seasonal isolation rates for each year from 2016 to 2019 are presented in Fig. 2b. Spring had the highest isolation rates except in 2017, when the highest isolation rate was in summer. Generally, the annual isolation rates of 2017 and 2018 were higher than those in 2016 and 2019 (p < 0.001), so the corresponding seasonal isolation rates were also relatively higher than those in 2016 and 2019.

### Distribution and prevalence of HA subtypes among LPAIVs

Among the 7567 samples, 974 (12.87%) were LPAIV-positive, with 890 single and 84 mixed infections. The numbers of the isolated subtypes and their percentages among species isolates are shown in Table 1. Of all LPAIV isolates, the H3 subtype accounted for the largest percentage, reaching 46.10%, followed by the H6 subtype (29.47%), the H9 subtype (9.03%), mixed infections (8.62%) and the H4 subtype (5.03%). H3, H4, H6, and H9 were the most prevalent subtypes, whereas H1 and H11 were isolated occasionally. Moreover, H1 and H4 were isolated mainly from ducks. Among the chicken isolates, which included four subtypes (H9, H3, H6 and H1), the H9 subtype was predominant, accounting for nearly half (46.48%), followed by H3 (23.94%). The duck isolates included six subtypes that are highly diversified. The H3 subtype was predominant and accounted for more than half (58.88%) of the isolates, followed by H6 (17.19%). The proportions of the other subtypes and mixed infections were all below 10%. The H11 subtype had only one isolate and was derived from ducks. Among the goose isolates, H6 was the most abundant subtype (77.56%), followed by H3 (10.24%), H9 (5.85%), mixed infection (5.37%) and H4 (0.98%).

**Table 1:** Distributions and percentages of the isolated HA subtypes.

Number	Isolated subtype	Chickens		Ducks		Geese		Total	
		Number	Percentage in chickens isolations (%)	Number	Percentage in ducks isolations (%)	Number	Percentage in geese isolations (%)	Number	Percentage in all isolations (%)
1	H1	1	1.41	15	2.15	0	0	16	1.64
2	H3	17	23.94	411	58.88	21	10.24	449	46.10
3	H4	0	0	47	6.73	2	0.98	49	5.03
4	H6	8	11.27	120	17.19	159	77.56	287	29.47
5	H9	33	46.48	43	6.16	12	5.85	88	9.03
6	H11	0	0	1	0.14	0	0	1	0.10
7	Mixed infections	12	16.90	61	8.74	11	5.37	84	8.62
Total		71		698		205		974	

**Figure 3:**
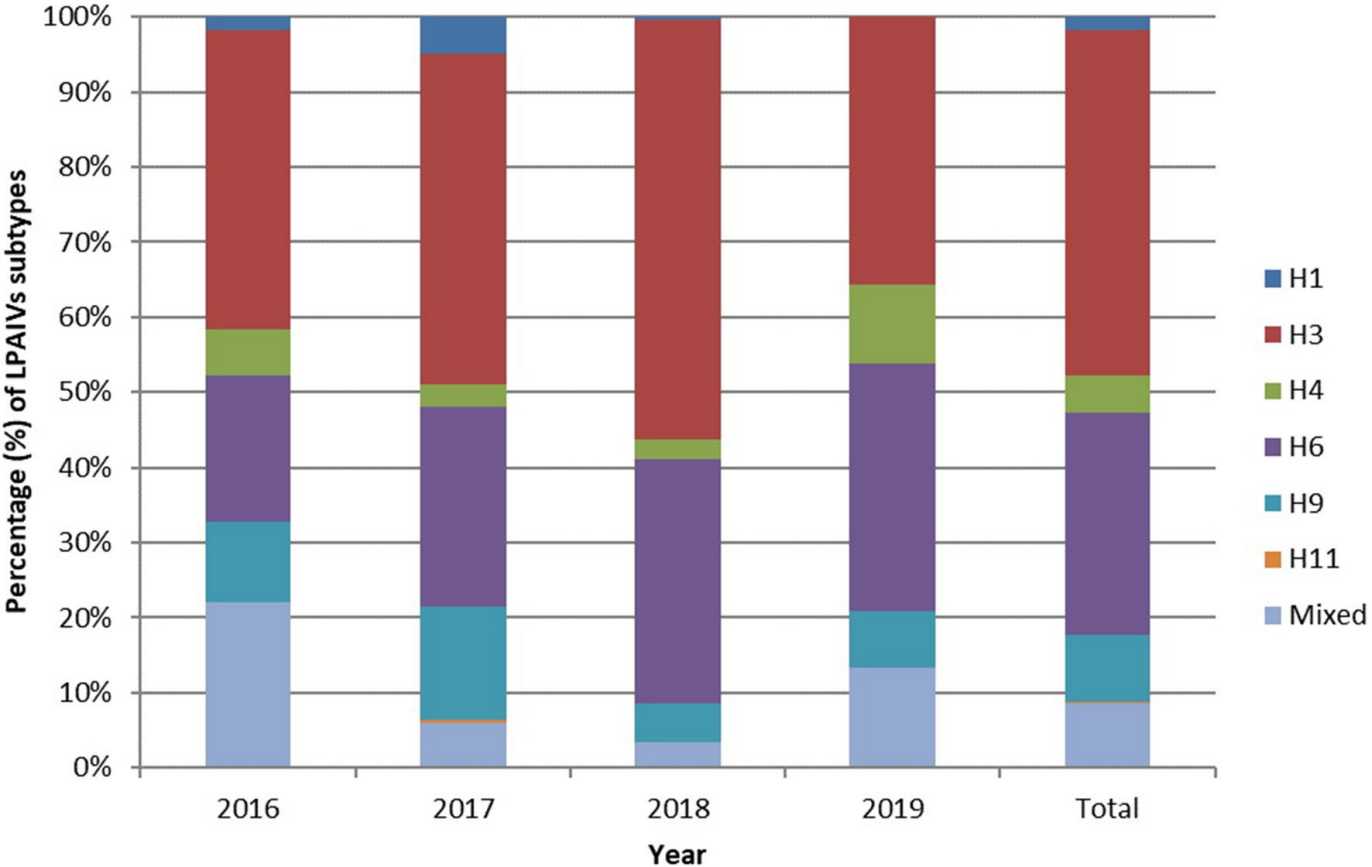
Percentage of the isolated HA subtypes from 2016 to 2019.

**Figure 4:**
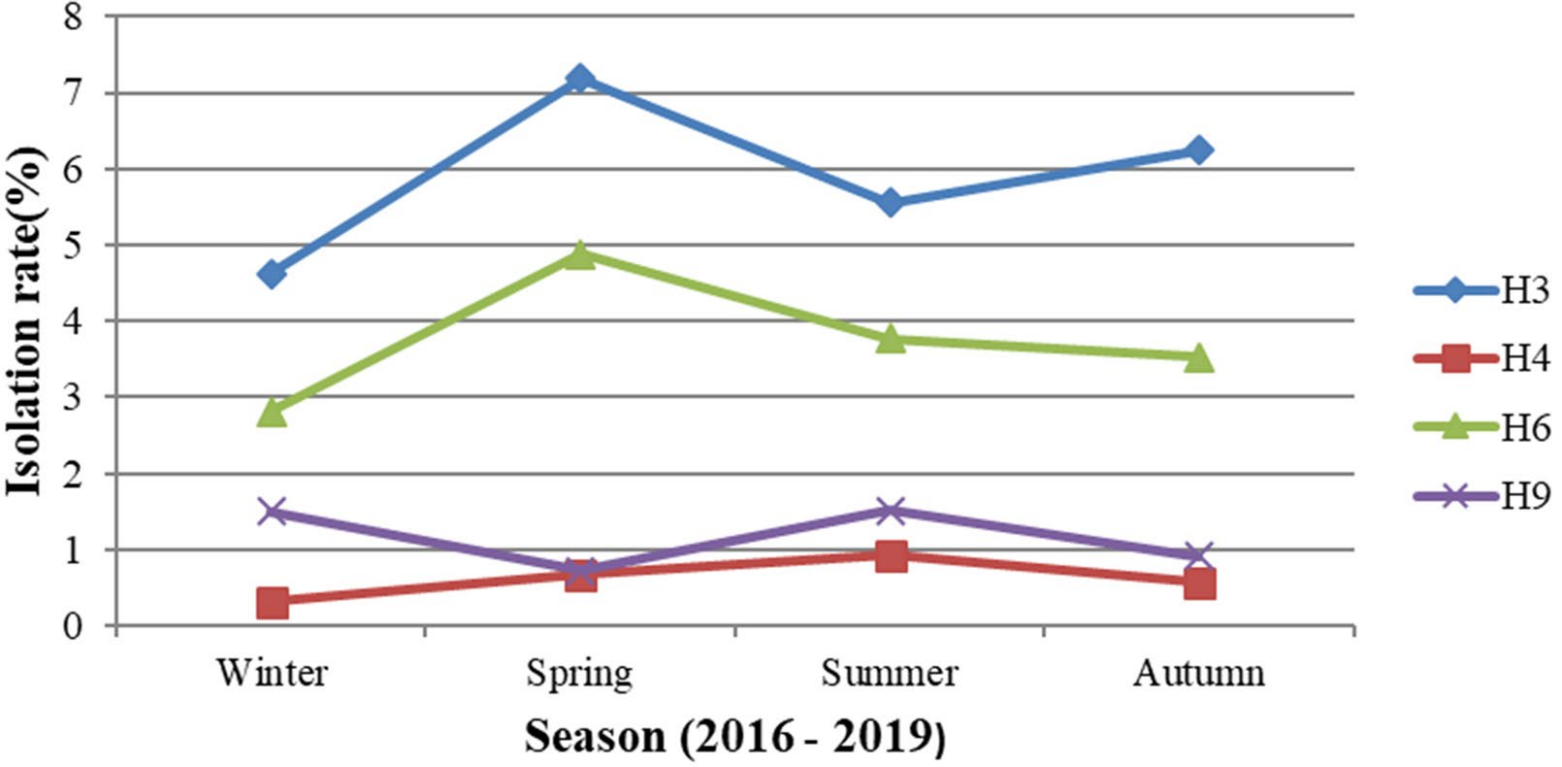
The isolation rates of the H3, H4, H6 and H9 subtypes in four seasons.

Figure 3 also shows the annual distributions of the isolated subtypes from 2016 to 2019. The trend of the annual distribution was similar to that of the four-year total distribution. In the four years, the H3 subtype had the highest percentage among LPAIV-positive samples each year, followed by the H6 subtype. The percentages of the H9 subtype were higher than those of H4 except in 2019. Twenty-four isolates were identified as the H4 subtype in 2019, accounting for 48.98% (24/49) of all H4 isolates. H3, H4, H6 and H9 were the main subtypes and could be identified in all seasons. Figure 4 reveals their isolation rates in four seasons. The highest isolation rates of H3 and H6 were both observed in the spring, and the H4 subtypes peaked in summer. Higher isolation rates of H9 were observed in winter and summer.

The HA and NA genes of the identified LPAIV isolates were sequenced and submitted to a BLAST search of the NCBI database. H1N2, H1N6, H3N2, H3N6, H3N8, H4N2, H4N3, H4N6, H4N8, H6N2, H6N6, H9N2 and H11N8 have been identified.

### The pattern of mixed infections

The percentage of mixed infections among the LPAIV-positive samples was 8.62% (84/974). Among the 84 samples with mixed infections, 12 were derived from chickens, 61 were derived from ducks and 11 were derived from geese (Table 1). Notably, the percentages of mixed infection cases were relatively high in 2016, decreased to relatively small percentages in 2017 and 2018, and then increased in 2019 (Fig. 3). Among the chicken isolates, mixed infections accounted for 16.90% (12/71). The percentages of mixed infections of ducks and geese corresponded to 8.74% (61/698) and 5.37% (11/205), respectively (Table 1).

As shown in Table 2, 84 mixed infection samples contained 22 combinations. Coinfections with two HA subtypes, which were the most common subtypes, comprised 17 combinations and resulted in 78 cases, accounting for 92.86% of the mixed-infection cases (78/84). Coinfections with three HA subtypes comprising 4 combinations and resulting in 5 cases were observed in chickens (2 combinations, 3 cases) and ducks (2 combinations, 2 cases). Coinfection with four HA subtypes comprised only 1 combination in 1 case and was observed in ducks. Among the cases of mixed infection, H3 + H4 coinfection was the most common and accounted for 33.33% (28/84) of all mixed infection cases and 27 cases were sourced from ducks.

**Table 2:** Mixed infections involving different HA subtypes in chickens, ducks and geese.

Number	Types of mixed infections	Chickens	Ducks	Geese	Total
1	H2 + H4	1	0	0	1
2	H2 + H6	0	0	1	1
3	H3 + H4	0	27	1	28
4	H3 + H6	0	0	2	2
5	H3 + H8	0	1	0	1
6	H3 + H9	0	5	0	5
7	H3 + H10	0	1	0	1
8	H3 + H11	1	9	1	11
9	H4 + H6	0	9	1	10
10	H4 + H13	0	1	0	1
11	H6 + H9	1	1	0	2
12	H6 + H11	0	1	5	6
13	H6 + H13	0	1	0	1
14	H9 + H10	1	0	0	1
15	H9 + H12	4	1	0	5
16	H9 + H16	0	1	0	1
17	H11 + H12	1	0	0	1
18	H1 + H2 + H10	0	1	0	1
19	H1 + H3 + H11	0	1	0	1
20	H3 + H4 + H9	1	0	0	1
21	H4 + H8 + H9	2	0	0	2
22	H3 + H10 + H11 + H14	0	1	0	1
Total		12	61	11	84

## Discussion

The term “highly pathogenicity avian influenza” (HPAI) generally refers to the strains that may induce an “intravenous pathogenicity index” (IVPI) greater than 1.2 or mortality rate over 75% in a defined chicken population during the specified interval of 10 days. Using this definition, all the HPAI strains isolated to date are of the H5 and H7 subtypes. However, viruses of these subtypes can also be of low pathogenicity. The World Organization for Animal Health (OIE) requires notification for all H5 and H7 subtypes, regardless of their pathogenicity, as they have the potential to mutate into HPAI viruses^7^. In this study, non-H5 and non-H7 AIVs are assumed to be LPAIVs. LPAIVs are a potential threat to humans, the prevalence of AIV infections should be monitored, and risks should be evaluated early. Prevention and control of LPAIVs should be continuously conducted in the long run. We analyzed the samples based on the data of three poultry species (chicken, duck, and goose), four years (2016, 2017, 2018 and 2019) and four seasons (winter, spring, summer and autumn) and recorded different subtypes of LPAIVs. We generated epidemiological data regarding the LPAIV isolation rates, the prevalent subtypes, and the percentages and distributions of subtypes and mixed infections. The increased isolation rates in 2017 and 2018 (Fig. 1) were mainly correlated with the increased isolation rates of ducks in the two years. The main reason was that the isolation rate of H3 subtype AIV in ducks significantly increased in 2017 and 2018; in addition, 13 out of 16 isolates of H1 subtype AIV were in ducks in 2017. Duck, as waterfowl, is more likely to interact with wild bird species, increasing the chance of AIV transmission across the wild bird-poultry interface, may lead to increased isolation rates in the two years. In Guangxi, spring had the highest isolation (Fig. 2a), and warm, humid and rainy conditions in spring may be favorable for the survival, growth and transmission of AIV. Compared to our LPAIV survey from 2012 to 2015^26^, mixed infections that decreased may be associated with expanding implementation of the “1110” strategy in live poultry markets. The 1110 strategy involves 1 daily cleaning, 1 weekly disinfection, 1 day of market closure every month and 0 live poultry stock overnight. Implementation of the strategy may decrease the isolation rates and mixed infection cases.

In the present study, the predominant subtypes in chickens, ducks and geese were H9, H3 and H6 (Table 1), respectively, in agreement with our previous survey results from 2012 to 2015^26^. Waterfowl are well known as a natural reservoir pool for AIV. The water resources of Guangxi are abundant and include many rivers, ponds and lakes etc., which provide good habitat for ducks, geese and other waterfowl. Poultry from LPMs is also derived from backyard farms, in which birds are typically raised as free-range scavengers with minimal to no biosecurity measures. These free-range birds may interact with wild bird species, sharing water, food, and habitat, increasing the chance of AIV transmission across the wild bird-poultry interface. In the present study, it is noteworthy that the proportion of H3 subtypes among the LPAIV samples all increased regardless of poultry species, which was especially obvious in ducks (Table 1). Domestic ducks serve as an ideal environment for the reassortment of H3 subtype influenza with other subtypes, which plays an important role in the ecology of AIV and may potentially be a threat to human health^27^, and in our study coinfection with H3 + H4 was the most frequently seen combination (Table 2). H3 subtype viruses have very diverse host, ranging from birds to various mammalican species, which may be easier to spread to other hosts by waterfowl and wild birds. Some H3N2 viruses of avian-origin were transmitted to dogs in South Korea, causing acute respiratory disease^28^. H3 subtype AIV from LPMs in China may contribute viral internal genes to H5N8 HPAIV^29^. H3 subtype AIV has become an important issue in emerging zoonotic infections and threats to public safety^30,31^. H6 subtype AIV can cause infection in mammals, for example, an H6N1 virus was isolated from a human with flu-like symptoms in Taiwan in 2013^17^, and an H6N6 virus was also detected in pigs in Guangdong Province in China in 2010^32^. The increased isolation of H3 in ducks and H6 in geese in our study provides an early warning and suggest that the epidemiolohical surveillance and genetic evolution analysis of H3 and H6 subtypes AIV should be enhanced and to find further information to support the prevention and control of AIV.

Chickens may play a key role in the evolution of the H9 subtype^18^. The H9N2 subtype was first identified from diseased chickens in Guangdong Province, China, in 1994 and became widespread among chickens, causing great economic losses due to reduced egg production and high lethality associated with coinfections with other avian pathogens^33^. Inactivated H9N2 commercial vaccines have been used in chicken flocks since 1998 in China, and the vaccines initially prevented outbreaks and transmission. However, in recent years, genetic alterations in H9N2 virus strains have reduced the effectiveness of vaccines, as H9N2 strains circulate continuously in vaccinated chicken flocks^19,34^. H9N2 is among the most common subtypes in chickens and can be easily isolated^33^. In our study, the H9 subtype accounted for nearly half of the chicken isolates (Table 1). Currently, H9 reassortants also comprise H5N2 and H5N6 subtypes^35,36^, in addition to the above mentioned H7N9 and H10N8 subtypes. The genes exchange or recombination of H9 strains with H5 and H7 etc. subtypes AIV may generate novel gene combinations resulting in emergence of HPAIV strains, which seriously threaten the healthy development of poultry industry and public health safety. The H9 reassortants cases continue to emerge, which may pose serious challenges to the prevention and control of AIV. The findings illustrated that the H9 vaccine needs to be updated.

Six subtypes with high diversity and complexity were isolated from ducks (Table 1). The duck is an important waterfowl species. Therefore, we collected more samples from ducks than from chickens and geese. The H4 subtype was mainly isolated from ducks in our study (Table 1). It has been reported that H4 combined with various NA subtypes circulates in LPMs incentral, eastern and southern China, and H4 and other subtypes (e.g., H3) have undergone complex reassortment events in domestic ducks^4,37–40^. Our results support the above observations, H4N2, H4N6 and H4N8 circulate, and mixed infections, including H4 are common in Guangxi Province (Table 2). The H4 subtype may have acquired the ability to infect, replicate and transmit in mammalian hosts^4,41^. Our results and other reports suggest that further investigations of the mechanisms of H4 AIV mutation and reassortment are important to prepare for potential pandemics.

Determining the prevalent subtypes of LPAIVs, the distribution and prevalence of these subtypes and the patterns of mixed infections and how they differ among subtypes may provide useful insight and guidance for the prevention and control of LPAIVs. Further epidemiological studies should continue to identify possible risk factors and to better understand the extent of AIV infection as well as potential transmission routes of AIVs. AIVs may occasionally infect humans exposed to infected poultry. Multisectoral coordination at the human-animal-environment interface is critical for zoonotic disease control^42^. Increasing surveillance in human and poultry populations is crucial for predicting and preventing the risk of new outbreaks. Efforts to collect samples not only from chickens, ducks and geese but also from other minor poultry species and humans as well as from the environment should continue in order to help monitor where AIVs circulate. Extensive surveillance of AIV infection and prevalence in LPMs is indispensable for clarifying the epidemic situation and infection landscape.

In conclusion, our study presents a comprehensive analysis of LPAIV infections in Guangxi, southern China from 2016 to 2019. Our results derived from an extensive 4-year effort provide essential surveillance data on the prevalence of different AIV subtypes in chickens, ducks and geese in LPMs, suggesting the need to enhance the monitoring and analysis of the genetic evolution and reassortment of H1, H3, H4, H6 and H9 subtypes AIV, especially for ducks and geese. Meanwhile, biosecurity measures should be improved in farms and LPMs to reduce the transmission of AIV and the occurrence of mixed infection. This information may lay a foundation for reducing the threat of novel or enzootic AIV subtypes of animal and human infections and could provide useful epidemiological data for efforts to effectively prevent and control influenza virus.

## Materials and methods

### Ethics statement

The present study was approved and conducted in strict accordance with the recommendations in the guide for the care and use of routinely sampled animals in LPMs by the Animal Ethics Committee of the Guangxi Veterinary Research Institute. Biological samples were gently collected from chickens, ducks and geese using aseptic cotton swabs. The birds were not anesthetized before sampling, and each sampled bird was observed for 30 min after sampling before being returned to its cage. All experiments were performed in accordance with the relevant guidelines and regulations. All methods were carried out in compliance with the ARRIVE guidelines.

### Sample collection

The epidemiological surveillance of LPAIVs in poultry was conducted at 20 selected LPMs in Guangxi Province, southern China. Among these LPMs, six representative LPMs in Nanning (the capital of Guangxi Province) were sampled in turn. Throat and cloacal swab samples from chickens, ducks and geese were collected from January 2016 to December 2019. We collected samples weekly. The sampling buffer was composed of sterile PBS (pH = 7.2) with penicillin (100 unit/ml), streptomycin (10 mg/ml), gentamycin (100 unit/ml) and nystatin (100 unit/ml). We collected throat and cloacal swabs and dipped them in sampling buffer. Swab throat and cloacal samples from the same birds were combined and considered to be one sample. The samples were kept at a low temperature (4 °C). After sampling, the collected samples were transferred to the laboratory for storage and testing. Swabs were cleaned, squeezed dry and discarded after adequate soaking. The swab solution was stored at − 80℃ until use.

### Virus isolation and hemagglutination inhibition test

The swab solutions were thawed and centrifuged in a sample tube at 3,000 × g for 10 min, and the supernatant was inoculated into10-day-old specific pathogen-free (SPF) chicken embryos (Beijing Merial Biology Company, Beijing, China) via the allantoic cavity. The chicken embryos were incubated at 35 °C after inoculation and were observed daily for the death of chicken embryos. The chicken embryos were chilled if they were still alive 5 days postinoculation, and then the allantoic fluids were collected and identified AIV by hemagglutination (HA) assay, which takes advantage of the tendency of HA protein of AIV to bind to red blood cells (RBCs) causing them to agglutinate. The isolates virus is serially-diluted and incubated with 0.5% RBCs to obtain HA titers. Samples with HA activity were subsequently identified HA subtypes by hemagglutination inhibition (HAI) assay, according to the World Health Organization (WHO) protocol (https://docplayer.net/15666727-Who-global-influenza-surveillance-network-manual-for-the-laboratory-diagnosis-and-virological-surveillance-of-influenza.html). When antibodies against a specific AIV HA protein bind to the antigenic sites on the HA protein, these sites become blocked and therefore unavailable for binding with RBCs. Briefly, the HAI assay was performed using 96-well microtitre plate, 4 hemagglutination units standardized antigen of isolate was mixed with serially diluted antiserum, and RBCs are then added to assess the degree of binding of the antibody to the HA molecule. The HAI titer was expressed as the reciprocal of the highest serum dilution in which hemagglutination was inhibited. All HI assays were performed in duplicate. A panel of reference antiseras were used against different HA subtypes (Table 3), Newcastle disease virus (NDV) and eggdrop syndrome (EDS).

**Table 3:** List of the primary strains used in the preparation of antisera of HAI assay.

Number	Subtype	Strain	HAI titer
1	H1	A/Chicken/Guangxi/GXc-1/2011(H1N2)	2^6^
2	H1	A/Duck/Guangxi/GXd-2/2012(H1N2)	2^7^
3	H2	A/Duck/HK/77/76(H2N3)	2^7^
4	H3	A/Duck/Guangxi/135D20/2013(H3N2)	2^8^
5	H3	A/Chicken/Guangxi/015C10/2009(H3N2)	2^7^
6	H3	A/Goose/Guangxi/139G20/2013(H3N2)	2^7^
7	H4	A/Duck/Guangxi/125D17/2012(H4N2)	2^7^
8	H4	A/Duck/Guangxi/101D18/2011(H4N6)	2^7^
9	H6	A/Duck/Guangxi/GXd-7/2011(H6N6)	2^8^
10	H6	A/Duck/Guangxi/GXd-2/2009(H6N2)	2^7^
11	H6	A/Goose/Guangxi/246G44/2016(H6N2)	2^6^
12	H8	A/Turkey/Ontario/6118/68(H8N4)	2^7^
13	H9	A/Chicken/Guangxi/066C10/2010(H9N2)	2^8^
14	H9	A/Chicken/Guangxi/137C2/2013(H9N2)	2^7^
15	H9	A/Goose/Guangxi/146G30/2013(H9N2)	2^8^
16	H10	A/Duck/HK/876/80(H10N3)	2^6^
17	H11	A/Duck/Guangxi/170/14(H11N2)	2^7^
18	H12	A/Duck/HK/862/80(H12N5)	2^6^
19	H13	A/Gull/Md/704/77(H13N5)	2^7^
20	H14	A/Mallard /Astrakhan/263/82(H14N5)	2^6^
21	H15	A/Shearwater/Western Australia/2576/79(H15N9)	2^7^
22	H16	A/Shorebird/Delaware/168/06(H16N3)	2^7^

### Gene sequencing and real-time RT-PCR

The single-infection isolates were further confirmed by plaque-purificationas previously described^43^, and then used for subsequent sequencing. Mixed-infection isolations were verified by real-time RT-PCR as previously described^44^. Viral RNA was extracted from viral stock fluid using an EasyPure Viral DNA/RNA kit (TransGen, Beijing, China) according to the manufacturer’s manual. cDNA was synthesized from viral RNA by reverse transcription with the 12-bp primer 5'-AGCAAAAGCAGG-3' as previously described^45^. PCR was performed using specific primers as described in previous research to obtain the full-length HA and NA genes^46^. The PCR products were purified with the TaKaRa Agarose Gel DNA Purification Kit Ver. 2.0 (TaKaRa, Dalian, China) and sequenced by Invitrogen of Guangdong Co., Ltd. A BLAST search was performed to compare the sequences against the nucleotide sequences of all known HA and NA genes of AIV in the GenBank database, and the HA and NA subtypes of the isolates were determined and verified.

### Statistical analysis

Data statistics were used to determine the differences in isolation rates of LPAIVs from different years, poultry species, seasons and subtypes. The results were analyzed using SPSS 22.0 software (IBM, Chicago, USA), the chi-squared test was used to analyze and compare the differences in these isolation rates, and p < 0.05 was indicative of a significant difference.
